# Skewed antibiogram of community-acquired urinary isolates and the therapeutic dilemma

**DOI:** 10.1186/cc14179

**Published:** 2015-03-16

**Authors:** A Chakraborty, S Roy, A Datta, A Kar

**Affiliations:** 1 Medica Superspecialty Hospital, Kolkata, India

## Introduction

Urinary tract infection (UTI) is one of the most common bacterial infections in humans. Gram-negative organisms being the most common causative agent, the rising prevalence of resistance to a number of antibiotics and more importantly the production of extended spectrum beta-lactamase (ESBL) by these organisms is a growing concern worldwide. As the scenario is no better in community isolates, the choice of empirical antimicrobials for such infections becomes a great challenge for the clinicians.

## Methods

In this retrospective observational study we aimed at knowing the prevalence of ESBL production by organisms causing UTI in the community and to study the antibiogram of such isolates. Urine samples from patients with suspected UTI in the community were cultured for uropathogen by routine microbiological methods and susceptibility testing was done on Microscan Autoscan 4 (Siemens).

## Results

Out of 527 isolates of Enterobactereaceae, 314 (59.58%) were ESBL producers from the community samples compared with 315 (67.30%) from hospital samples, with *Escherichia coli *being the most commonly isolated pathogen. *Enterobacter *spp. showed highest prevalence (80%) of ESBL production from the community samples. Among the ESBL producing strains from the community, the sensitivity to ciprofloxacin, levofloxacin and nitrofurantoin was 18%, 21% and 44% respectively while in the non-ESBL producers the sensitivity rates were 52%, 51% and 73% respectively.

## Conclusion

Organisms producing the ESBL phenotype present with an added possibility of being resistant to other broad-spectrum antimicrobial agents which are commonly prescribed in the community to empirically treat such infections. This makes the choice of empirical antibiotic much more challenging in the community, drawing errors in judgment. A possibility of frequent overcorrection lies on the other side of the coin. This study also shows the possible need for empirical institution of class I carbapenems as one of the treatment options and outpatient parenteral antimicrobial therapy.

